# Simulation-based learning in postgraduate critical care and anaesthesia nursing: an interview study from postgraduate nurses’ perspectives

**DOI:** 10.1186/s12912-025-03336-x

**Published:** 2025-07-01

**Authors:** Carina Sjöberg, Pether Jildenstål, Mona Ringdal, Torben Nordahl Amorøe, Viktoria Sjöstedt, Carl-Johan Cederwall

**Affiliations:** 1 Department of Medicine & Health Sciences, Lund University, Sölvegatan 19 Hus E plan 15 Box 117, Lund, 223 62 Sweden; 2 Institute of Health and Care Sciences, Sahlgrenska Academy, University of Gothenburg, Goteborg, Sweden; 3 Department of Anaesthesiology, Surgery and Intensive Care, Sahlgrenska University Hospital, Goteborg, Sweden; 4 Department of Anaesthesiology and Intensive Care, School of Medical Sciences, Örebro University Hospital, Örebro University, Örebro, Sweden; 5 Department of Anesthesiology and Intensive Care, Skane University Hospital, Lund, Sweden; 6 Department of Molecular and Clinical Medicine, Institute of Medicine, Sahlgrenska Academy, University of Gothenburg, Gothenburg, Sweden; 7 Region Västra Götaland, Department of Research, Education & Innovation, Sahlgrenska University Hospital, Simulation Centre West, Gothenburg, Sweden; 8 Region Vastra Gotaland, Department of Research, Sahlgrenska University Hospital, Development, Education and Innovation, Gothenburg, Sweden; 9 Department of Anesthesiology, Surgery and Intensive Care, Sahlgrenska University Hospital, Mölndal, Sweden; 10 Faculty of Nursing and Health Sciences, Nord University, Bodø, Norway

**Keywords:** Simulation-based learning, Postgraduate critical care and anaesthesia nursing

## Abstract

**Background:**

Simulation-based learning is used to educate students in anaesthetics and critical care. At the postgraduate level, the aim is to foster an understanding of the connection between theory and practice and prepare nurses for the clinical environment. In this context, we created a new wherein we integrated simulation-based learning with clinical learning activities e.g. before start of clinical practice, during and after end of clinical practice. The postgraduate nurses were educated and instructed by university teachers from the postgraduate program and clinical active tutors, critical care nurses and registered nurse anaesthetists from the simulation center. This study aimed to explore the experiences of postgraduate nurses with simulation-based learning and its impact on their overall learning outcomes.

**Method:**

This is a descriptive qualitative interview study with a purposeful sample of postgraduate nurses with experience with simulation-based learning during clinical practice. The data were analysed using inductive and thematic analysis.

**Results:**

We identified two themes ‘pros of simulation-based learning’ and ‘cons of simulation- based learning’ describing opportunities and obstacles related to the postgraduate nurses’ experience of simulation-based learning. ‘Pros of simulation-based learning’ included the subthemes *‘learning through scenarios facilitates preparedness for clinical practice*,*’ ‘learning via reflection gives you time to think over your actions’ and ‘Learning from tutors is valuable and highly regarded* ‘Cons of simulation based learning’ included the subthemes *‘it is difficult to accept the simulation situation and environment*,* ‘the design of the scenarios and technical conditions constitutes a barrier’ and ‘Unfamiliar groups interfere with learning.*

**Conclusion:**

This study shows that simulation-based learning activities promoted postgraduate nurses learning especially later in the education. Competence required for critical care nurses and registered nurse anesthetists is situational and competence specific, theoretical background and clinical experience are therefore required to promote progression in learning.

**Supplementary Information:**

The online version contains supplementary material available at 10.1186/s12912-025-03336-x.

## Background

Simulation-based education is a general term for practical exercises conducted under safe conditions across various settings. This approach allows learners to gain hands-on experience and develop their skills in a controlled environment, reducing the risks associated with real-world practice. In an academic context, simulation-based training (SBT) [1] and simulation-based learning (SBL) [2] are two methodologies that leverage simulated environments for educational purposes. Despite their similarities, they have distinct foci and applications [1, 2]. SBT is a structured and often formalised approach to training where participants engage with advanced simulators to practice specific skills and procedures. It is commonly used in professional and vocational training environments, such as aviation, health care and military settings [3–5]. The simulators used in SBT are often sophisticated, replicating real-world tools and scenarios to provide an immersive experience [6, 7]. SBL, on the other hand, is a broader educational strategy that utilises simulation as a tool for experiential learning, often within an academic or pedagogical framework [8, 9]. SBL encompasses a wide range of activities, from skills training, role-playing exercises to - virtual simulations [10, 11]. The goal of SBL is to facilitate deeper understanding, critical thinking and reflective learning. It emphasises the learning process over the mastery of specific skills, encouraging learners to explore scenarios, make decisions and experience the consequences of those decisions in a safe environment [9, 12, 13].

In summary, while both SBT and SBL employ simulation as a core component, SBT is more focused on skill acquisition and competency in performing specific tasks through repetitive practice and assessment, often in professional training contexts. SBT for health care professionals (HCPs) is well established to improve patient safety, especially by improving teamwork and communication when using the Crisis Resource Management (CRM) instrument [4]. In contrast, SBL emphasises the broader educational process, fostering critical thinking and experiential learning through a variety of simulated experiences. SBL has gained prominence in postgraduate nursing education and is usually used as an effective and valuable tool to train students in an academic setting in preparation for entering a clinical environment [14]. Slavinska et al. [15] suggests that simulation-based education in a risk-free environment should serve as a distinct intermediate stage between theoretical learning and clinical practice in medical and healthcare curricula. Notably, postgraduate nurses have experience and clinical competence, unlike undergraduate nursing students, and thus have completely different needs in the context of SBL regarding developing the clinical competence that is required of them as critical care nurses (CCNs) and registered nurse anaesthetists (RNAs) [16, 17]. Postgraduate nurses entering the education environment with different clinical backgrounds and experiences constitutes a pedagogical challenge that must also be considered in the context of SBL. Instructors must be aware of this and consider that the postgraduate nurses may have different learning strategies, divergent learning experiences and different perceptions and beliefs about their own competence [13].

During SBL, the postgraduate nurses take part in a dynamic scenario and deal with several problems arising during the simulated procedure. This step is designed to provoke the development of their technical and/or non-technical skills [18]. In both SBT and SBL, can the simulator be very similar to reality or, alternatively be designed specifically for practicing skill acquisition through part-task training [10]. Reflection and debriefing are central in SBT and SBL and can be carried out in different ways during and after simulation [19].

The education and responsibilities of CCNs and RNAs vary between countries. In Sweden, RNAs work independently in perioperative care and in collaboration with the anaesthesiologist. CCNs work independently with nursing care and in collaboration with the intensivist [20]. CCNs and RNAs in the same context may in other countries have similar duties or work less independently as an assistant to a physician. Specialist nurses in Sweden are responsible for and make decisions regarding prioritising care in high-technology environments such as the operating theatre and the intensive care unit. Specialist nurses work in interprofessional teams most of their working time [20]. Well-functioning teamwork is therefore crucial for providing optimal health care, though it is also challenging [21]. High-technology environments are a new context, with advanced tasks, teamwork and increased responsibility for the postgraduate nurses. Clinical practice is central for them to get familiar with this new context and develop suitable skills [22]. Quality of healthcare is inherently linked to the quality of healthcare professionals’ education and the extent of their professional development. Consequently, from two perspectives the necessity of simulation-based education to ensure high-quality healthcare and the imperative to provide high-quality education—simulation-based learning plays a critical role in enhancing both clinical competence and patient outcomes [15]. Influencing factors for the quality at clinical practice include the patient population served by the department, the pedagogical and didactic skills of the staff and, most importantly, their knowledge of the latest evidence from the literature [23]. It is challenging to offer an equal education for postgraduate nurses in clinical practice, the student’s vulnerability and the mentor’s pedagogical competence and learning strategy are factors to consider creating opportunities for individual learning [24]. SBL for student’s nurse anesthetists is beneficial to educate them to manage anesthetic emergencies has good results but is also very costly [25]. For graduate nurses enrolled in an intensive care course can multiple exposures to high-fidelity simulations improve the learning outcomes in the in managing virtual critically ill patients’ care needs [26].

This study is part of a two-year collaborative learning project between a university and one health care region. The project was initiated by the health care region to investigate whether it was possible to replace two weeks of clinical practice with simulations. From the university’s perspective, clinical learning activities could be more evidence-based, and academic discussions could be integrated within the clinical context. Additionally, a safer environment was established, ensuring that an equivalent education could be provided from a legal standpoint.

In the project, we intergraded SBL with clinical learning activities before, during and after clinical practice. The clinical learning activities were evidence-based to a greater extent, e.g. literature recommendations in syllabi, to guarantee the highest learning outcomes. University teachers from the postgraduate program and clinical active tutors, CCNs and RNAs teach and instruct in the SBL activities. It is therefore of value to describe postgraduate nurses’ views about integrated SBL with clinical learning activities before, during and after clinical practice and in what time it would be perceived as most effective for them.

## Method

### Aim

This study aimed to explore the experiences of postgraduate nurses with SBL and its impact on their overall learning outcomes.

### Design

This study was designed with a qualitative approach to evaluate a two-year SBL intervention for postgraduate nurses. Data were collected through semi-structured individual interviews and data analyses were then performed inductively with thematic analysis based on Braun and Clarke [27].

### Setting and participants

This study was conducted at a university within the education program for CCNs and RNAs. In Sweden, becoming a registered nurse requires three years of undergraduate-level higher education with 180 credits, including a Bachelor of Science in nursing. To become a CCN or RNA, one year of study at the postgraduate level is required, which includes a postgraduate diploma in specialist nursing: a one-year master’s degree (60 credits) [28].

Purposeful sampling was used to ensure the recruitment of postgraduate nurses with experience with the SBL activities in the project. The project was first presented orally to the postgraduate nurses during the presentation of the project. Written information was provided via e-mail to all in the one-year CCN and RNA course during a two-year project period (50 students/year). This ensured that the potential respondents met the inclusion criteria to participate in the study. Those who agreed to participate were given further information about the study from the researchers. They were invited to read the information and were given time to decide whether to participate. They were then contacted again, and interview times were arranged. Before the interviews started, the participants gave their written consent.

### The model

**Fig. 1:**
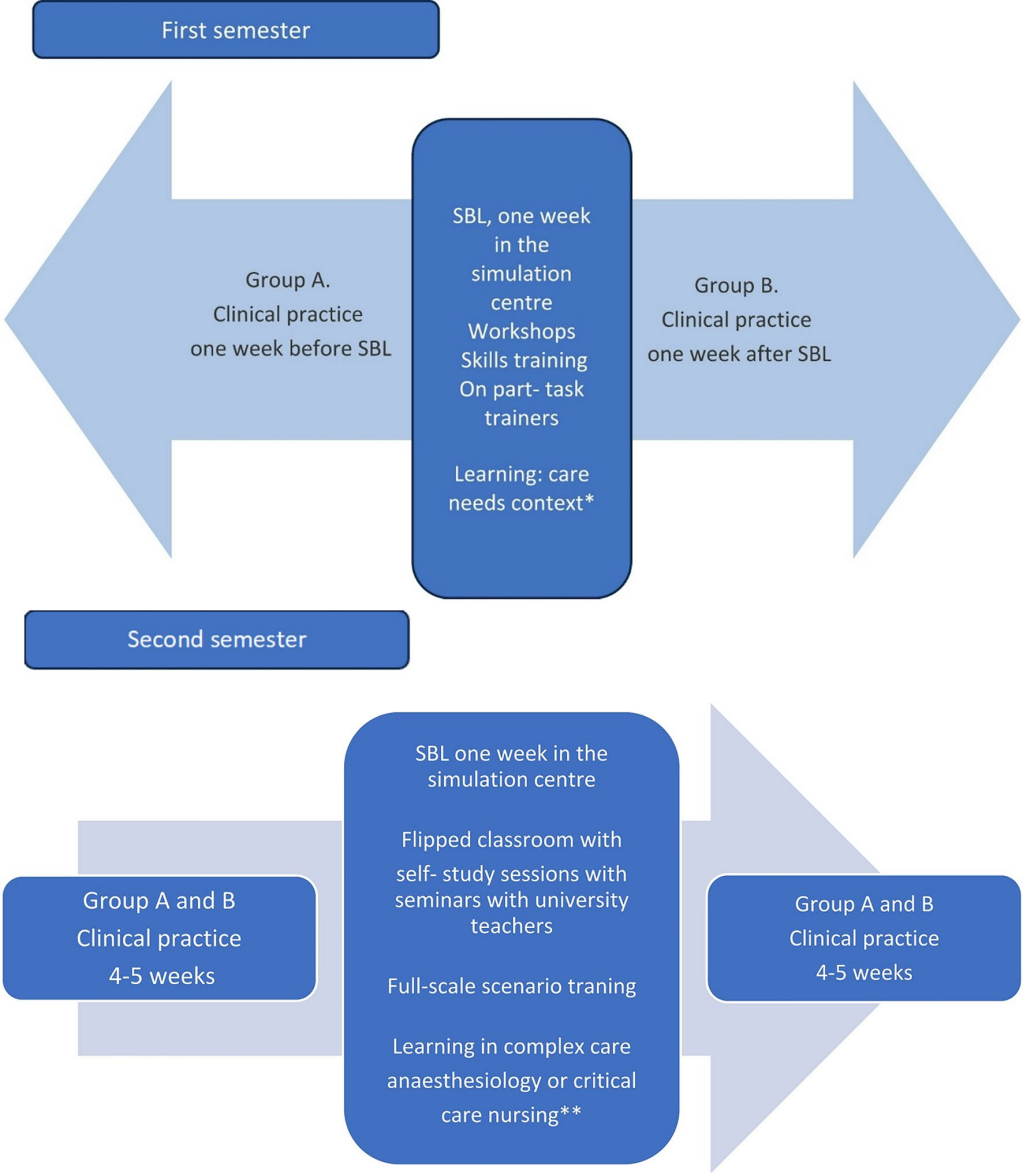
Flowchart of simulation-based learning (SBL). *The course objective in the first semester of the anaesthesia or critical care curriculum was care needs in high-technology units. The learning objectives included knowledge and understanding, competence and skills and judgement and approach. **The course objective in the second semester was deepening complex care in anaesthesiology and nursing in critical care, aiming for progression in the learning objectives of knowledge and understanding, competence and skills and judgement and approach. Example in intensive care, scenarios with patients who have undergone thoracic and neurosurgery. Example in anesthesia scenarios with adverse events, pediatric anesthesia and extubation

In the project, SBL was used as a learning activity in two postgraduate nursing programs, one in critical care nursing and one in anaesthetic nursing. Each program consists of theory and two weeks of clinical practice in the first semester and eight weeks of practice in the second semester. The SBL activities were carried out at a simulation center where practical and communicative skills are trained in simulated near-realistic environments in efficient and patient-safe ways. The HCPs working at the simulation center have solid knowledge and extensive experience working with simulations. In this environment, learning situations can be adapted and tailored to suit participants’ specific circumstances and needs. University teachers from the postgraduate program designed learning activities from the syllabi. Two weeks of clinical practice were changed to SBL activities, which were designed together with HCP working at the simulation center. The model is presented in Fig. 1.

The SBL sessions were led by HCP, working partly at the simulator center and partly in the clinic as clinical nurses and tutors. The tutors were ordinarily responsible for the postgraduate nurses’ learning during clinical practice. Before the project started, the tutors received two days of formal education under the supervision of experienced facilitators. The full-scale scenarios were complex and rare situations in anaesthesia and intensive care.

### Data collection

During spring after SBL in the second semester in 2022 and 2023, 14 individual semi-structured interviews were conducted digitally on Zoom. The participants were informed in advance that the interviews would focus on their experiences with SBL. The interviews were conducted by VS. All interviews were recorded and transcribed verbatim by a professional transcriber. An interview guide with modified questions based on Gibbs [29] was designed and used to get a description of the postgraduate nurses’ experiences. Interview guide se supplementary file. The interview guide started with the opening question: ‘Can you tell me about your experiences with the simulation-based learning activities?’ followed by further questions about the simulation situations. The interviews ranged from 17 to 56 min in duration. Data saturation was reached after 14 interviews, at which point no new information emerged and the interviewees were beginning to repeat remarks made in previous interviews [30].

### Data analysis

Data were analysed inductively using thematic analysis for identifying patterns (themes) within data according to Braun and Clarke [27]. In the first phase, the interview transcripts were read by CS and CJC and reread to gain familiarity with the content and obtain a sense of the whole. Initial coding was performed by CS in NVivo (version 14) and organised into groups. The initial codes were discussed amongst the authors CS and CJC and confirmed by PJ and MR. The codes were then compared and combined to form themes and subthemes. These groupings were reviewed and discussed by all the authors until a consensus was reached. In the next phase, the themes were reviewed and refined in relation to each coded data extract and to the entire data set (i.e. all data used for the analysis). Finally, the findings were summarised and quotes were selected to elucidate the themes and subthemes. An example of the analysis process is shown in Table 1.

**Table 1:** Examples of the analysis process

Data extract	Code	Sub theme	Theme
Have sort of processed the theoretical knowledge that we´ve been given, but then things happened where I thought, ´Ah, now I get what you mean with the beep. ´	Understand theoretical	Learning through scenarios facilitate preparedness for clinical practice	**Pros for learning**
You pick up things like, wow, that’s was great how she reacted there, ‘or ´I´ll remember that for next time. ´	Learning from peers	Learning via reflection gives you time to think over your actions	
I haven ´t felt like anyone was afraid to ask questions or scared to say something that they might think is stupid.	Open learning climate	Learning from tutors is valuable and highly regarded	
I thought it was weird with the mannequins, like, ´Oh, is this for real? Are we pretending now, or not?	Unreal situations	It is difficult to accept the simulation situation and environment	**Cons for learning**
Because you have to pretend to change things since the simulation lungs, for example, can´t handle higher pressure.	Simulator limits	The design of the scenarios and technical conditions constitutes a barrier	
In the class, you haven´t talked to everyone, so there were many people I had never spoken to before, and then you´re suddenly thrown into that situation.	Uncomfortable in the group	Unfamiliar groups interfere with learning	

### Ethics

The ethics of the study were reviewed by the Swedish Ethical Review Authority, Uppsala department 2 medicine. Dnr 2021-06634-01. The study was conducted in accordance with the Declaration of Helsinki. All participants were informed about the study verbally and via written information and provided written consent to participate.

### Findings

The age of the postgraduate nurses in the sample (*n* = 14) varied between 26 and 55 years with a mean age of 36.8 years. Their work experience in healthcare varied between 2 and 37 years with a mean of 14,2 years. Work experience as nurses varied from 2 to 25 years with a mean of 9,5 years. Based on the interview findings we identified two themes, *pros for learning* and *cons for learning* these were supported by six subthemes related to the postgraduate nurses’ experience of SBL (Table 2).

**Table 2:** Subthemes and theme

**Themes**	Pros for learning	Cons for learning
**Subthemes**	Learning through scenarios facilitates preparedness for clinical practice Learning via reflection gives you time to think over your actions Learning from tutors is valuable and highly regarded	It is difficult to accept the simulation situation and environment The design of the scenarios and technical conditions constitutes a barrier Unfamiliar groups interfere with learning

### Pros for learning

The environment in which SBL activities take place, combined with various forms of reflection involving tutors and peers, contributes to positive learning outcomes for postgraduate nurses. The application of theoretical knowledge in practical contexts fosters learning progression, while active engagement in teamwork and the assumption of diverse roles within the team further enhance understanding of the clinical setting. These positive learning outcomes are exemplified through the identified subthemes.

#### Learning through scenarios facilitates preparedness for clinical practice

The postgraduate nurses reported that the environment supported their learning by being safe and permissive. The novel experience was that the scenarios were conducted in a way that enabled them to feel free to make errors, talk and ask questions about what they did not understand.

*You get to practice it beforehand*,* uh*,* on a dummy*,* so it’s like… there’s no danger*,* no one dies* (Interview no. 11).

Everyone was in the same situation and the postgraduate nurses perceived fewer demands than in clinical practice, which contributed to their learning. Moreover, the scenarios were perceived as de-dramatised and therefore not as serious situations as in reality, which reduced their fear of making mistakes and drawing incorrect conclusions. The skills training included repositioning an endotrachal tube, adjusting ventilator and settings, handling different catheters and drainage. All were perceived as contributing to gaining knowledge that is difficult to learn theoretically. In other words, the scenarios enabled the theoretical knowledge to become comprehensible when it became part of a context. It was pointed out that they were persons who learned practically. The practical component thus becomes fundamental and indicates readiness for clinical practice. Some of the scenarios were uncommon situations, which the postgraduate nurses highly appreciated having the opportunity to practice. Moreover, questions could have arisen during clinical practice, but there was reportedly no time and space to understand and get answers in the clinic.

*We had days divided between seminars in the morning and simulations in the afternoon*,* or the other way around. It worked really well because we went through articles related to the cases we were about to review. I thought that setup was perfect* (Interview no. 8).

The opportunity to repeat the scenario a second time was perceived as positive. After having detected mistakes and after reflecting in the debriefing, the postgraduate nurses were able to do it again with a much better result. Sometimes the scenarios were considered challenging, with the postgraduate nurses getting into situations where they got tunnel vision. Nevertheless, they were perceived as positive learning situations because they gained knowledge on how they could use certain strategies to prevent this in the future.

*This is the subject we need to practice because it was clear that ‘we don’t know this.’ So*,* you also gain valuable insight into what you don’t know* (Interview no. 5).

Teamwork was also valuable to practice in different roles around the patient. Specialist nurses are responsible for the leadership standing at the patient’s head with responsibility for the patient’s free airway. Or drug administration and making sure that orders from physicians are carried out. Or with responsibility for correct equipment and assistance. Practicing teamwork from these different perspectives and using the CRM principles for communication meant that the postgraduate nurses were aware of the whole context and the importance of the different roles. Moreover, they described that they sometimes learned from peers’ actions in the scenario. The situation also gave them the insights that when you work in a team you are never alone, and that communication is significant.

#### Learning via reflection gives you time to think over your actions

Reflection was a vital component of the postgraduate nurses’ learning progression. By reflecting with peers and tutors-clinical nurses, they deepened their knowledge. This, in turn, increased their self-confidence. Reflection was both planned and spontaneous, depending on the situations that arose in the scenario. Reflection was done during the scenarios with interruptions and in debriefings after the scenarios. In addition, the postgraduate nurses spontaneously reflected with their peers. They noted that time for reflection was central. When the scenario was interrupted for reflection, they could take a step back and ask themselves the following questions: What is happening? Why is it happening? What can I do? What are my options?

*That you have the time to actually reflect a bit and have time to think about what’s happening with the patient*,* without harming anyone.* (Interview no. 1)

The possibility of doing such a reflection in a scenario without any patient getting hurt was seen as very instructive. In the debriefing after the scenarios, they said the reflection developed their knowledge when they were able to understand how things were connected. Through reflection, they were also able to understand how to manage the different roles in the team. Moreover, the experience helped them develop as individuals with increased self-awareness. Discussing their thoughts and daring to talk about problems that arose contributed to learning. Peers were highlighted as important for learning; using their different experiences to discuss and ask each other questions was considered very valuable.

*I think this whole simulation-based training is*,* um… I mean*,* you learn a lot about yourself too*,* how you act in certain situations*,* which I can take with me into my future profession.* (Interview no. 5)

Consulting their peers meant that they could confirm that they were thinking correctly and give each other constructive feedback. When they had gained clinical experience, they were able to further develop their learning in reflections.

#### Learning from tutors is valuable and highly regarded

The tutors were very important for the learning process. The participants noted that information about and the structure of the learning activities was clear and the tutors were interested, engaged, willing to teach and therefore inspiring.

*They are very skilled at asking knowledge-based questions: How did you think in this situation? Instead of giving answers*,* they turn it back to you: You did this. How did you think? What was it based on?* (Interview no. 7).

Tutors contributed with an approach through which postgraduate nurses felt they could be themselves and comfortable when asking questions. By answering questions, the tutors confirmed that they understood situations correctly. Questions from an individual postgraduate nurses perspectives also contributed to the learning process, which in turn gave rise to new perspectives. The experience that emerged was that it was easier to ask questions in the simulation context than in clinical practice. The tutors were not seen as teachers; they were perceived as clinically active nurses, and this was of great importance. Tutors were great respected and their knowledge was validity and valuable for them, and not being in an assessment situation was seen as an advantage.

*We’re here to teach you*,* not to assess you.” It helps you feel less pressured* (Interview no. 14).

### Cons for learning

Discouraging emotions like embarrassment and scepticism before the scenario was considered to hinder the participants’ learning. They also highlighted the importance of the composition of the group, the design of the scenario, the technical conditions as well as how realistic the simulation environment was for learning outcomes. Barriers to learning are illustrated by the subthemes.

#### It is difficult to accept the simulation situation and environment

Initially having low expectations, the postgraduate nurses found acting in the simulation environment difficult. Their perception was that they were going to engage in playacting as in a theatre and not practise for real. Some thought it got easier over time while others continued to be uncomfortable in the situation. Talking to the simulator manikin was considered troublesome. They described the problem as not being able to take the situations seriously and feeling uncomfortable, which made it difficult to act correctly, for example when an alarm rang in a monitor.

*Oh*,* I didn’t think of that” or “I should have known this” – you become a bit blank when it’s not really real; you might have acted differently if it was real*,* but now you didn’t take it as seriously*,* like when the monitor beeped*,* because you knew it wasn’t real* (Interview no. 13).

This was problematic, and it was obvious that it affected their learning when they did not progress in their thinking and reasoning as a result. Some felt that someone else would solve the problem – that there was a person in the control room who could help them if they did not solve the task. While they felt uncomfortable in the simulation situation and the environment, they were aware of the risk of achieving no progress in their learning.

*I think that if you don’t fully commit to it*,* you won’t get much out of it either*,* and you might not take it as seriously. If you feel like*,* “No*,* this won’t give me anything*,*” if you already go in with that mindset*,* then you won’t get much out of it* (Interview no. 5).

#### The design of the scenarios and technical conditions constitutes a barrier

Some postgraduate nurses reported that the time allocated for the scenarios was too short. The scenarios were also interrupted when they just got in the flow in their work and wanted to continue to learn more. Sometimes the interruption came at inappropriate times, such as when the situation involved helping free a patient’s airway and an action such as intubation was needed. When such situations were interrupted and the participants were invited to share their feelings in a debriefing, conflict and ambivalence arose.

*You’re supposed to carry the feeling you had when you were interrupted*,* and I could almost feel a sense of not being finished. It was almost a bit frustrating because you noticed that the patient couldn’t breathe and wanted to help with that. But then we had to go and reflect*,* and it felt a bit like*,* “Hey*,* ABCD*,* A comes before R* (Interview no. 10).

Sometimes the time for the practical exercise before the scenario was too short. Another problem was that the degree of difficulty of the scenarios and the content was perceived as uneven, with too much repetition of basic topics. There were also deficiencies in theoretical preparation. The postgraduate nurses felt like failures if they were not adequately prepared for the scenario. It became obvious to them that they lacked knowledge. The debriefing after the scenarios was considered valuable, but they thought that it was too extensive in relation to what they learned. Moreover, the debriefings were also sometimes perceived partly as too structured but also unprofessional. However, it was obvious that debriefings were generally viewed as important. At one point, the debriefing did not happen, which was perceived as the learning not being complete. The postgraduate nurses also reported flaws in the simulation environment and said the scenarios could be unrealistic. This could have occurred due to missing relevant lab results and adequate equipment in cabinets and a lack of medicines. Learning could also have been limited by the shortcomings and limitations of the technology.

*I think one reason we didn’t move forward was… when we did the A-E assessment of the patient and when we felt the mannequin*,* they had to repeat to us because you can’t feel if the patient is warm or cold* (Interview no. 6).

Sometimes, it was unclear if the equipment was actually broken or if it was meant to be broken in the current scenario. Participants also reported that the simulator could limit learning, as the manikin could not change colour and temperature, and that there was a lack of volume and pressure values during mechanical ventilation.

#### Unfamiliar groups interfere with learning

The group was considered important for learning. Initially, the postgraduate nurses did not know each other, which was perceived as an obstacle. Being thrown into a new situation with others was experienced as a challenge. They also noted that generational differences could influence attitudes, with being older possibly correlating with a greater fear of making mistakes. Not knowing what was going to happen made them feel a little nervous, so it took some time for them to get into the ‘role.’

*The first three simulation days*,* we were in the same groups. And on the last day*,* we switched*,* which felt a bit unsettling because you had gotten comfortable with the group you had been working with* (Interview no. 4).

Some of them had previous experience with certain situations in the scenarios, which led them to point out that the experience of encountering the same situation with a group they did not know made it totally different.

*CRM – we had an introduction to it beforehand and we use it a lot at my job*,* so I’m very used to it. But then it’s always interesting to think*,* “Yeah*,* I know this*,*” but you can’t do CRM by yourself. The whole group needs to be on board. So*,* I’m quite used to using it*,* or I think I use it*,* but everyone needs to be of that mindset* (Interview no. 12).

## Discussion

To our knowledge, this is the first study describing postgraduate nurses’ experiences regarding the advantages and disadvantages of SBL and its overall impact on their learning. The tutors were highly valued as an essential resource, while the learning scenarios and peer reflections were also considered particularly beneficial. Additionally, prior experiences, individually perceived learning outcomes, and the pedagogical approach played significant roles in learning. However, learning could be hindered by the limitations of the simulated environment and the artificial nature of the scenarios.

The tutors and a judgment-free environment facilitated learning in SBL settings by providing structured introductions, respectful interactions, and an atmosphere that encouraged speaking up. This aligns with the concept of psychological safety described by Daniels et al. [31], which is a core element for high-quality learning activities throughout all phases of simulation-based education. Similarly, Bello et al. [32] emphasized in their review that psychological safety is crucial for fostering sustainable learning environments, ultimately leading to optimal patient outcomes, long-term staff retention, and improved economic conditions in perioperative settings. Nordahl Amorøe et al. [33] highlighted the importance of resilience, enabling capability training and improvements in the quality of care. Overall, the SBL environment significantly enhances postgraduate nurses’ sense of security, self-esteem, and confidence, thereby promoting effective learning [10].

Tutors with clinical experience were also regarded as valuable resources, as they dedicated time to addressing questions arising from clinical practice. It is well established that insufficient time in clinical settings limits opportunities for student feedback and reflection [22]. Reflection can range from descriptive to deeper levels, incorporating an iterative process involving experience, understanding, and action [34]. This study emphasized the importance of time for reflection and its central role in learning progression. Creating a psychologically safe environment for reflection is even more critical than the specific structure of reflection or debriefing, as highlighted by both nursing students and faculty members in a study by Turner et al. [35]. A safe learning environment within familiar peer groups was also perceived as beneficial in this study. Psychological safety is similarly emphasized by Skedsmo et al. [13] in their study on postgraduate palliative care nursing students.

The effects of SBL are particularly pronounced for students unfamiliar with the clinical context and tasks, as demonstrated by Chernikova et al. [14] in their meta-analysis on SBL in higher education. These findings align with the results of the present study. Postgraduate nurses reported that engaging in various roles within SBL activities provided valuable insights and knowledge, allowing them to integrate theoretical concepts into clinical practice. This integration of theory into real-world scenarios is a fundamental goal of SBL for both undergraduate and postgraduate nursing education [10, 16].

However, challenges related to skepticism toward SBL were noted. Some postgraduate nurses experienced emotions such as embarrassment, discomfort, and low expectations regarding the scenarios and their learning outcomes. Those who failed to recognize the potential of SBL tended to limit themselves, thereby reducing their learning opportunities. Similar findings were reported by Skedsmo et al. [13] in a study on postgraduate palliative care nursing students’ experiences with SBL focused on communication skills. Repeated exposure to SBL activities may help mitigate these challenges by increasing familiarity with the learning method [16, 26].

Nevertheless, students will always approach a simulated scenario differently than a real-life situation [10]. Therefore, thorough theoretical and practical preparation before participation in SBL is essential. When postgraduate nurses perceived scenario introductions as inadequate, they experienced feelings of failure, incompetence, and an inability to succeed. Additionally, the perceived realism of the scenario was influenced by its level of difficulty, technical malfunctions, and equipment limitations. Poorly designed, unrealistic scenarios and insufficient technology may, therefore, negatively impact learning outcomes [36].

Some postgraduate nurses reported that scenarios were too short and that debriefing sessions overemphasized emotional responses rather than focusing on skill acquisition. While reflection was acknowledged as important, practical skill development was prioritized. Participants expressed a preference for practicing entire processes within a scenario.

The findings of this study indicate that a standardized pedagogical approach may not be suitable for both postgraduate nurses and other healthcare professionals, given the differences in their educational needs and clinical contexts.

The conventional simulation modality described in the 2021 International Nursing Association for Clinical Simulation and Learning (INACSL) Healthcare Simulation Standards of Best Practice™: Simulation Design [37] cannot be directly applied as an empirical reference for SBL in postgraduate nursing education. This is because the basic pedagogical approach and learning objectives outlined in the 2021 INACSL standards differ from those regulated in SBL syllabi. Another challenge is that experienced simulation educators often lack structured pedagogical development and a deeper theoretical understanding of teaching and learning, relying instead on self-confidence in their role [38].

In this study, postgraduate nurses appreciated SBL activities incorporating active learning and a flipped classroom approach—an educational model that emphasizes student-centered learning rather than traditional lectures [12]. Student-centered learning involves peer collaboration and reflective discussions. It is crucial that tutors recognize their role in facilitating or hindering student-centered reflection [39]. Reflection, in various forms, was perceived as essential for learning and progression in SBL. Flipped classrooms in graduate nursing education have been shown to enhance performance and are generally well received by students, although further research is needed to establish definitive evidence [40]. The flipped classroom model is particularly well suited for SBL as it fosters critical thinking, teamwork, and problem-solving in real-world scenarios. However, adherence to the pedagogical principle that students must prepare in advance is essential [12]. The findings of this study are valuable in addressing the challenge of providing adequate clinical training opportunities in anaesthesia and intensive care, fields in which multiple professional categories require clinical experience. These results may contribute to further research and discussions on how SBL can be effectively integrated into clinical practice.

## Strengths and limitations

The transferability [41] of the findings of this study is uncertain, as it depends on different types of education for CCNs and RNAs concerning the variation in work tasks worldwide. However, despite this and other cultural differences, there are likely similarities in learning when combining theory with practice. Reflection, learning from tutors, learning activities and the learning environment all tend to be meaningful. There was an age variation amongst the postgraduate nurses but a homogeneity in gender. However, they had different prior experience levels, which may have contributed to rich descriptions and further enhanced the credibility [41] of the results. The dependability [41] is strengthened by the fact that the same author conducted all the interviews, but it may have been negatively affected because the data collection was done over two years, even if the of interview guide partially strengthens. The analysis process was interactive, with input from and discussions amongst all authors until consensus was reached to strengthen confirmability [41]. We have been transparent in the description of our methods and analysis and present quotations in the results to facilitate transferability.

## Conclusion

This study demonstrates that postgraduate nurses find simulation-based learning (SBL) valuable, particularly when supported by theoretical and contextual understanding. The findings suggest that SBL is most effective when introduced later in the educational program, aligning with learners’ clinical experience. The rationale behind this is that the competencies required for CCNs and RNAs are both situational and highly specific. As such, the progression of postgraduate nurses’ learning in SBL is closely linked to their theoretical knowledge and clinical experience. It has thus been essential for their learning and development to engage in educational activities that combine evidence-based theoretical knowledge with skills training, including the use of part-task trainers and full-scale scenario-based training with reflection, all within the same context. Given the limited research in this area, further studies are needed to explore the pedagogical challenges associated with educating postgraduate nurses from diverse clinical backgrounds and experiences.

## Electronic supplementary material

Below is the link to the electronic supplementary material.

Supplementary Material 1

## Data Availability

The qualitative data upon which this analysis was conducted are not publicly available due to ethical concerns regarding confidentiality of participants. Further, consent was not obtained from participants to share information from interview transcripts with third parties not involved in the research and the ethical approval for this study don’t permit the sharing of such information.
