# Supplementary material for: Simulation-based learning in postgraduate critical care and anaesthesia nursing: an interview study from postgraduate nurses’ perspectives
Source: BMC Nurs. 2025 Jul 1;24:816. doi: 10.1186/s12912-025-03336-x (PMC12211888; doi:10.1186/s12912-025-03336-x)
Supplement: Supplementary file 1 — Supplementary Material 1 [file 12912_2025_3336_MOESM1_ESM.docx]

Interview guide

Opening question:

Can you tell us about your experiences with the simulation exercises?

Follow-up questions:

What happened during the simulation that affected you?

What thoughts and feelings were evoked by this event?

What is the most important knowledge that the simulation has given you?

How can you use what you learned in your profession as a specialist nurse?

What significance do you think what you have learned has for patient safety?

Are there any other situations that you think would be appropriate to simulate?

What has contributed to or hindered your learning?

Modified questions based on:

Gibbs G. Learning by doing: a guide to teaching and learning methods. London: FEU; 1988.
